# Early-life microbial contact with adult hens modulates cecal microbiota and mitigates host responses to lipopolysaccharide-induced stress in broilers

**DOI:** 10.1186/s40104-026-01511-x

**Published:** 2026-10-10

**Authors:** Haoran Zhao, Ans De Ceuster, Muhammad Zeeshan Akram, Luke Comer, Elena Fako, Matthias Corion, Nadia Everaert

**Affiliations:** Nutrition and Animal-Microbiota Ecosystems Laboratory, Department of Biosystems, KU Leuven, Heverlee, Belgium

**Keywords:** Broiler, Early-life colonization, Gut microbiota, Intestinal barrier, Laying hen, LPS challenge, Tryptophan metabolism

## Abstract

**Background:**

In commercial production, the absence of adult hens disrupts natural gut colonization, forcing chicks to rely on environmental microbes and potentially compromising the ecological succession essential for health. This study investigated whether early-life adult hen microbial contact could optimize the intestinal community and metabolite profiles, thereby mitigating lipopolysaccharide (LPS)-induced immunological stress in broilers. Accordingly, 128 one-day-old broilers were assigned to control or adult hen microbial contact groups. Hens were removed on D 10, and LPS was administered on D 18 and D 20, resulting in a 2 × 2 factorial design.

**Results:**

Adult hen microbial contact did not affect body weight or ileal histomorphology on D 10 or D 21. However, it reshaped the alpha diversity (*P* < 0.05) and beta diversity (*P* < 0.05) of the cecal microbiota, enriching beneficial genera such as *Bacteroides*, *Prevotellaceae*, *Rikenellaceae*, *Muribaculaceae*, and *Megasphaera*, although these were not primarily established through direct adult hen transfer. These microbial alterations were accompanied by increased production of cecal volatile fatty acids (*P* < 0.05) and altered tryptophan metabolism (*P* < 0.05), characterized by the conversion of serum tryptophan to indoleacetic acid. In the ileum, the adult hen microbial contact upregulated (*P* < 0.05) genes associated with mucosal barrier integrity (*MUC13*, *CLDN2*) and antioxidant defense (*SOD2*, *FOXO1*), while independently downregulating (*P* < 0.05) hepatic genes involved in redox regulation and autophagy (*GRX5*, *LC3-1*). LPS challenge induced a pronounced (*P* < 0.05) antioxidant imbalance in the ileum, characterized by the downregulation of *CAT* and *NRF2*, together with elevated expression of pro-inflammatory cytokines (*IL8*, *IFNA*, *TNFA*), compromising barrier function (*MUC13*, *CLDN2*). Concurrently, the LPS challenge increased (*P* < 0.05) the expression of hepatic genes involved in redox regulation and autophagy (*GRX5*, *LC3-1*), consistent with a systemic stress response. Notably, microbial contact attenuated the LPS-induced reduction in valeric acid (*P* < 0.05), suggesting a protective effect on microbial metabolite production under immunological stress.

**Conclusion:**

Adult hen microbial contact did not improve growth performance or ileal histomorphology, but shaped the early-life gut microbiota and associated metabolite profiles, contributing to improved resilience to subsequent immune challenges in broilers.

**Supplementary Information:**

The online version contains supplementary material available at https://doi.org/10.1186/s40104-026-01511-x.

## Introduction

The broiler chicken intestine harbors a complex microbial community that plays an important role in regulating growth performance, nutrient metabolism, and immune maturation [1]. However, this microbiota is not present from birth. Under natural conditions, early colonization is largely driven by maternal transmission, where chicks acquire pioneer microbes through direct contact with the adult chicken and its environment [1–4]. This maternal seeding is critical for establishing the intestinal microbiota, as the timing and sequence of microbial arrival shape community assembly [5]. In commercial production systems, this process is disrupted because chicks are hatched separately from adult hens under highly controlled hygienic conditions [6–8]. Recent evidence further indicates that direct vertical transmission of gut bacteria is limited under commercial production conditions, with most hen-associated taxa remaining undetected or occurring at low frequencies in young chicks [9]. Consequently, microbiota acquisition relies primarily on environmental sources, which may delay the establishment of key commensals and result in a less stable microbial community [1]. As a result, young chicks may be more susceptible to pathogen colonization and impaired immune development [6, 10]. Therefore, enhancing early-life microbial exposure has emerged as a promising strategy to improve gut microbiota development and health in commercial broilers.

Among the proposed strategies, fecal microbiota transplantation has been shown to accelerate gut microbiota maturation in broilers [6, 11, 12]. However, this approach provides only an acute microbial inoculation rather than sustained microbial exposure. Therefore, sustained exposure to the microbiota of adult hens may more effectively promote microbiota establishment. However, changes in microbial composition alone do not fully explain how early-life microbial interventions affect host physiology, as many microbiota-mediated effects are exerted through microbial metabolites [6]. Most studies focus primarily on taxonomic shifts [6], while the metabolite-mediated mechanisms linking microbiota development to host immune function remain poorly understood. For instance, volatile fatty acids (VFAs) derived from microbial fermentation enhance local immune responses and inhibit pathogen colonization [13, 14]. Concurrently, tryptophan (TRP) metabolism is pivotal for host immunity. The gut microbiota metabolizes TRP into indole derivatives like indole-acetic acid to strengthen the mucosal barrier [15], whereas host metabolism during immune stress converts TRP to kynurenine [16]. Crucially, these locally produced metabolites enter the systemic circulation, allowing serum metabolic profiles to serve as vital indicators of the dynamic crosstalk between gut microbial activity and systemic immune status [15, 17]. However, the impact of early-life microbial contact on these circulating metabolites remains poorly understood.

Lipopolysaccharide (LPS) derived from the Gram-negative bacterial cell wall is a major pathogen-associated molecular pattern that activates innate immunity [18]. In commercial farming systems, broilers are commonly exposed to Gram-negative bacteria, making LPS a frequent inducer of immune stress [18–20]. LPS can disrupt redox balance and trigger an acute inflammatory response, making its application a commonly used experimental model for studying immune stress in broilers [18, 21]. Given the widespread exposure to LPS in farming environments, it remains unclear whether early-life microbial contact can offer protective effects. Such intervention may act by reshaping gut microbiota composition and altering microbiota-derived metabolites, including VFAs and TRP metabolites. Whether these changes are sufficient to enhance intestinal barrier function, regulate immune-related gene expression, and ultimately alleviate LPS-induced systemic inflammatory injury has yet to be determined. Addressing this knowledge gap may provide a physiological and practical strategy to improve gut health and immune resilience in commercial broiler production.

We hypothesized that early-life adult hen microbial contact promotes the establishment of a more mature cecal microbiota, reshapes microbial metabolite profiles, and consequently improves intestinal barrier function and immune regulation, thereby enhancing broiler resilience to LPS-induced immune stress. To test this hypothesis, ISA Brown laying hens were selected as adult donor chickens to provide newly hatched broilers with early exposure to a mature microbial community, followed by an LPS-induced immune stress challenge. By integrating data on cecal microbiota, microbial metabolites, and host gene expression, this study aims to mechanistically understand how adult hen microbial contact can improve broiler health under commercial production conditions.

## Materials and methods

### Animals, housing, and experimental design

Prior to the start of the broiler experiment, four clinically healthy 61-week-old ISA Brown laying hens were obtained from a commercial farm in Belgium and distributed in four pens for 10 d to naturally seed the environment. ISA Brown laying hens were selected as adult donor chickens with a mature and stable gut microbiota to provide early microbial exposure to newly hatched broilers. Subsequently, a total of 128 one-day-old male Ross 308 broilers with an initial body weight of 43.0 ± 0.3 g were purchased from a commercial hatchery (Belgabroed N.V., Merksplas, Belgium). Chicks were randomly assigned to two groups: a control group (C) and a microbial contact group (H). Each group comprised four pens, with 16 chicks per pen. In the H group, one hen was co-housed in each pen but physically separated from the chicks by a wire partition. To facilitate microbiota transfer, the locations of the hens and broilers within each pen were swapped once daily at approximately 10:00, and the procedure was completed within approximately 5 min. The hens were removed from the pens on D 10. All pens were initially bedded with fresh wood shavings and equipped with heat lamps to maintain optimal brooding temperatures. Importantly, no additional wood shavings were added during the rearing period. Feed and water were provided ad libitum via standard feeders and drinkers. To minimize diet-induced variation in the microbiota, the laying hens were provided with the commercial broiler starter diet during the environmental seeding and microbial contact periods. The broilers received the same starter diet from D 0 to D 10 and were switched to the grower diet after the hens were removed (Additional file 1: Table S1).

Before LPS administration on D 18, body weight did not differ between the microbial contact and control groups (678 ± 19 g vs. 683 ± 15 g, *P* = 0.850). The study was then transitioned into a 2 × 2 factorial design by physically dividing each of the original pens (both C and H) into two compartments. Half of the birds in each pen were challenged with lipopolysaccharide (LPS), while the other half received a sham injection. Specifically, LPS (*Escherichia coli* O55:B5, L2880, Sigma Aldrich, St. Louis, MO, USA) was dissolved in phosphate-buffered saline (PBS) to a concentration of 1 mg/mL. Challenged birds received an intraperitoneal injection of 1 mg LPS/kg body weight, while control birds received an equivalent volume of sterile PBS. This resulted in four final experimental groups, each group had 4 replicates, with 7 chicks per replicate (four pens for each treatment): control + PBS (CC), microbial contact + PBS (HC), control + LPS (CL), and microbial contact+ LPS (HL).

### Sample collection

On the day of chick arrival, fecal samples were collected from the four hens, snap-frozen, and stored at −80 °C for subsequent DNA extraction and microbial sequencing. On D 10 and D 21, prior to individual sampling, body weight was measured at the pen level. Subsequently, eight birds per treatment group (2 chicks per pen) were randomly selected and euthanized for sampling. All subsequent analyses were performed using these eight biological replicates per treatment group. Following electronarcosis, blood was collected from the jugular vein prior to decapitation. Serum was collected after centrifugation (2,000 × *g*, 15 min, 4 °C) and stored at −20 °C. All tissue collection was performed in a dedicated room to prevent cross-contamination. Cecal content was collected, snap-frozen in liquid nitrogen, and stored at −80 °C. For histological analysis, a 3-cm segment of the mid-ileum was excised, gently flushed with ice-cold PBS, and fixed in 4% formalin. Additionally, on D 21, the mid-ileal tissue and the left liver lobe were collected, snap-frozen, and stored at −80 °C for further analysis.

### Ileal histomorphology

The ileum was selected for histomorphological analysis because it is a primary site of mucosal immune activity and barrier function, which are central to the host responses to microbial intervention and LPS challenge evaluated in this study [22, 23]. The morphological analysis of ileal segments followed the methodology described in previous studies [24]. The fixed ileal segments were dehydrated, cleared, and embedded in paraffin. Tissue sections were cut, deparaffinized in xylene, rehydrated, and subsequently stained with Alcian Blue-Periodic Acid Schiff to visualize the ileal architecture. The stained sections were visualized at 20 × magnification (NDP.view2 software, Hamamatsu Photonics, Hamamatsu, Japan). The villus height (VH) and crypt depth (CD) were measured. The villus height to crypt depth ratio (V/C) was also calculated.

### DNA extraction, 16S rRNA sequencing, and bioinformatics workflow

Microbiota analysis was performed according to our previous study [11]. Specifically, total DNA was isolated from broiler cecal samples (D 10, D 21) and adult hens’ feces utilizing the QIAamp PowerFecal Pro DNA Kit (Qiagen Benelux B.V.). Following quality control on a NanoDrop 2000, the 16S rRNA V3–V4 region was targeted. Sequencing was conducted by Novogene Co., Ltd. on an Illumina NovaSeq 6000, yielding 250 bp paired-end reads (around 50,000 reads per sample). The raw sequencing data were processed in QIIME2 (v2024.9), involving demultiplexing and DADA2 denoising to generate ASVs. Rarefaction was applied consistent with Schloss [25] for alpha and beta diversity analyses. Taxonomy was assigned via a Naive Bayes classifier trained on the SILVA 138 database with 99% identity [26, 27]. Final QIIME2 artifacts were exported to R (v4.2.3) for downstream analysis.

For taxonomic composition analysis, the top 5 genera were selected independently for each experimental group (Donor, C, and H) based on mean relative abundance, with low-abundance genera grouped into an ‘Others’ category. On D 10, ASVs exclusively shared between the Donor and H groups, but absent in the C group, were identified and operationally classified as ‘Donor-Derived’. The proportion of these donor-derived ASVs relative to the total abundance of their respective genera within the H group was subsequently calculated. Venn diagrams illustrating these intersections and abundance plots were generated using the ggVennDiagram and ggplot2 packages in R. The Chao1 index and Shannon index was calculated to evaluate alpha diversity. Group differences on D 10 were analyzed using a linear mixed-effects model with microbial contact as a fixed effect and pen as a random effect. On D 21, microbial contact, LPS challenge, and their interaction were included as fixed effects, with pen as a random effect. Beta diversity was visualized via Principal Coordinate Analysis (PCoA) based on Bray-Curtis distances, and group differences were assessed using PERMANOVA. We employed Linear Discriminant Analysis Effect Size (LEfSe) via the microbiomeMarker package to identify differential taxa. Only genera satisfying both an LDA score > 4.0 and a False Discovery Rate (FDR) adjusted *P*-value < 0.05 (Benjamini-Hochberg method) were retained for visualization.

### Volatile fatty acid concentration

VFA concentrations in cecal content were quantified using an HP 6890 Series GC System, as previously described [26]. Briefly, 250 mg of cecal digesta was mixed with 100 µL of 2-methylhexanoic acid (internal standard), followed by the addition of 200 µL of 25% NaCl and 200 µL of 9.2 mol/L hydrochloric acid. Organic acids were extracted with 800 µL of diethyl ether. The mixture was centrifuged at 3,800 × *g* for 5 min at 4 °C. The resulting supernatant was transferred to a vial containing 0.2–0.3 g of activated anhydrous sodium sulfate (drying agent) and centrifuged again (3,800 × *g*, 6 min, 4 °C) prior to GC analysis.

### Serum tryptophan metabolites

Serum aliquots (250 μL) were deproteinized by adding 100 μL of 60% perchloric acid and 100 μL of internal standard solution (3-nitrotyrosine, 60 ng/μL). The volume of each sample was adjusted to 750 μL with ultrapure water in 1.5-mL microcentrifuge tubes. The mixture was vortexed for 10 s, incubated on ice for 5 min, and centrifuged at 14,000 × *g* for 10 min at 4 °C. The supernatant was filtered through a 0.22-μm nylon filter into autosampler vials and stored at 4 °C until analysis. Analysis was performed on a Shimadzu UHPLC system equipped with a photodiode array (PDA, SPD-M40) and fluorescence detector (RF-20Axs). Separation was achieved on a Shim-pack XR-ODS column (100 mm × 3.0 mm, 2.2 μm; 40 °C) protected by a GVP-ODS guard column. The mobile phase consisted of 10 mmol/L sodium acetate buffer (pH 4.8) and acetonitrile, delivered at a flow rate of 0.7 mL/min using gradient elution. The injection volume was 30 μL. The PDA monitored the 190–800 nm range with primary detection at 360 nm, while fluorescence detection was set to an excitation of 280 nm and emission of 370 nm. Standard references of TRP, kynurenic acid, and indoleacetic acid were purchased from Sigma-Aldrich. Stock solutions were prepared in DMSO (kynurenic acid, 3.9 mg/mL), sodium phosphate buffer (TRP, 4.5 mg/mL), or ethanol (indoleacetic acid, 4 mg/mL) and stored at −20 °C. Calibration curves were established by correlating peak areas to analyte concentrations, using retention times and UV spectral data for identification.

### RNA extraction

Total RNA was extracted from the ileum and liver using the ReliaPrep™ RNA Miniprep System (Promega, Madison, WI, USA). Subsequent quantification was performed on a NanoDrop 2000 (Thermo Fisher Scientific, Waltham, MA), and integrity was visualized on a 1% agarose gel.

### Primer design and validation

A panel of 93 ileal (Additional file 1: Table S2) and 89 hepatic (Additional file 1: Table S3) genes related to physiological processes was selected. Gene selection was guided by previous evidence showing that these genes and pathways are responsive to gut microbial modulation and LPS-induced immune stress, thereby enabling a comprehensive assessment of physiological processes potentially affected by microbial contact, including barrier function, nutrient transport, antioxidant and mitochondrial responses, immune regulation, programmed cell death, and tryptophan metabolism [6, 20]. Exon-spanning primers (< 30 nt, amplicon < 150 bp) were designed via NCBI Primer-BLAST or sourced from the literature. Primer validation was conducted as previously described [24]. Briefly, efficiency and specificity were evaluated on a QuantStudio 6 Real-Time PCR instrument (Thermo Fisher Scientific) using three-fold serial dilutions of a pooled cDNA sample. Specificity was further confirmed via melting curve analysis and agarose gel electrophoresis for single-band verification.

### Reverse transcription and preamplification

Complementary DNA was synthesized from 50 ng of total RNA utilizing the Standard BioTools Reverse Transcription Master Mix (Standard BioTools, South San Francisco, CA, USA) according to the manufacturer’s instructions. For specific target amplification, a primer mixture was prepared by combining 1 µL of each 100 µmol/L forward and reverse primer and adjusting the final volume to 400 µL with Tris-EDTA buffer to reach a final concentration of 250 nmol/L for each individual primer. This primer mixture was then combined with Fluidigm PreAmp Mastermix and DNase-free water to form a pre-amplification mix. Subsequently, 3.75 µL of this pre-amplification mix was combined with 1.25 µL of the cDNA template. Thermal cycling was performed as follows: initial denaturation at 95 °C for 2 min, followed by 14 cycles of 95 °C for 15 s and 60 °C for 4 min. To eliminate unincorporated primers, samples were treated with Exonuclease I (New England Biolabs, MA, USA). The resulting products were diluted 10-fold in TE buffer and stored at −20 °C prior to analysis.

### High-throughput qPCR

High-throughput quantitative PCR was conducted using the BioMark HD system (Standard BioTools) with 96.96 Dynamic Array integrated fluidic circuits (IFCs), following the established protocols [24]. To determine primer efficiency, standard curves were generated from a three-fold serial dilution of a pooled cDNA sample containing equal aliquots 10 µL from all biological replicates. The reaction mixtures were prepared as follows: the sample mixture contained 2.5 µL of 2 × SSoFast EvaGreen Supermix with low ROX (Bio-Rad) and 0.25 µL of 20 × DNA Binding Dye. The assay mixture comprised 2.5 µL of 28 × Assay Loading Reagent and 2.25 µL of 1 × TE buffer (TEKnova, Hollister, CA, USA). The thermal cycling protocol consisted of an initial denaturation at 95 °C for 60 s, followed by 30 cycles of 96 °C for 5 s and 60 °C for 20 s. Fluorescence data were acquired using SBI Real-Time PCR software (version 1.0.2). The stability of candidate reference genes was evaluated using NormFinder [28]. In ileal tissue, seven candidates (GAPDH, 28S, RPS7, TBP, UB, YWHAZ, B2M) were assessed, and TBP, RPS7, and GAPDH were identified as the most stable combination. In hepatic tissue, six candidates (B2M, β-actin, GAPDH, UB, YWHAZ, TBP) were tested, with YWHAZ, β-actin, and UB selected for normalization. Relative gene expression was calculated using the Pfaffl method [29], with normalization to the geometric mean of the 3 tissue-specific reference genes.

### Statistical analysis

Data normality was first assessed using the Shapiro-Wilk test in R. Following the confirmation of normality, data from D 10, including body weight, histomorphological parameters, VFA concentrations, and TRP metabolites were analyzed using a linear mixed-effects model with microbial contact as a fixed effect and pen as a random effect (*Y*= *μ* + *T* + *P* + *ε*), where *Y* is the dependent variable, *μ* is the overall mean, *T* is the fixed effect of microbial contact, *P* is the random effect of pen, and *ε* is the residual error. For D 21, data were analyzed using a linear mixed-effects model with microbial contact, LPS challenge, and their interaction as fixed effects, while pen was included as a random effect (*Y* = *μ* + *M* + *L* + *M* × *L* + *P* + *ε*), where *M* is the fixed effect of microbial contact, *L* is the fixed effect of LPS challenge, and *M* × *L* represents their interaction. When a significant interaction between the fixed effects microbial contact and LPS was observed, pairwise comparisons were performed using Tukey’s post hoc test. Statistical significance was defined as *P* < 0.05. Results were visualized using GraphPad Prism 9. For ileal and hepatic gene expression, *P*-values were adjusted for multiple comparisons using the Benjamini-Hochberg method, and adjusted *P*-values < 0.05 were considered statistically significant. The overall variation in gene expression profiles was visualized via Principal Component Analysis (PCA), and group differences were statistically assessed using PERMANOVA. Furthermore, the expression patterns of genes exhibiting significant main effects were visualized using heatmaps based on Z-scored group means. Additionally, Spearman’s rank correlation analysis was conducted to explore associations between differentially abundant genera (identified by LEfSe) and metabolites. Correlations were considered significant if the absolute correlation coefficient |*R*| > 0.5 and adjusted *P* < 0.01.

## Results

### Body weight and ileal histomorphology indices

**Fig. 1:**
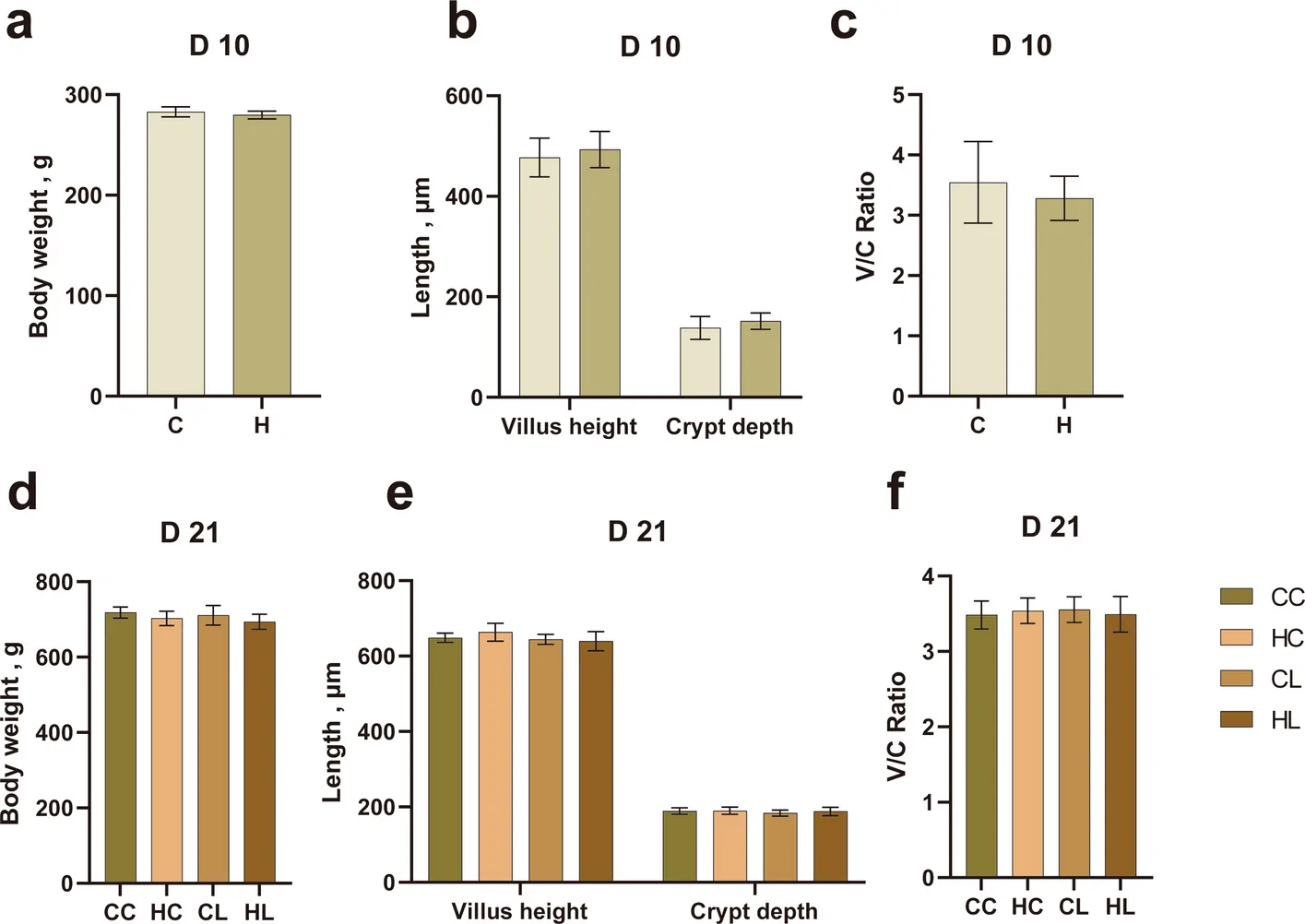
Effects of microbial contact on body weight and ileal histomorphology. **a**, **d** Body weight of broilers. **b**, **e** Villus height and crypt depth of the ileum. **c**, **f** Villus height to crypt depth ratio (V/C ratio). C: control, H: microbial contact, CC: control + PBS, HC: microbial contact + PBS, CL: control + LPS, HL: microbial contact + LPS. Data are presented as mean ± SEM, *n* = 8 biological replicates. On D 10, data were analyzed using Student’s *t*-test. For D 21, a two-way ANOVA was employed to evaluate the main effects of microbial contact and LPS challenge, as well as their interaction. Significance was defined as *P* < 0.05

On D 10, body weight in group H did not differ significantly from that in group C (Fig. 1a; *P* > 0.05). There were no significant differences in ileal VH, CD, or V/C ratio between the two groups (Fig. 1b and c; *P* > 0.05).

On D 21, neither the microbial contact nor the LPS challenge had a significant main effect or interaction on body weight, VH, CD, or V/C (Fig. 1d–f; *P* > 0.05).

### Microbiota analysis

#### Microbial composition and donor contribution

**Fig. 2:**
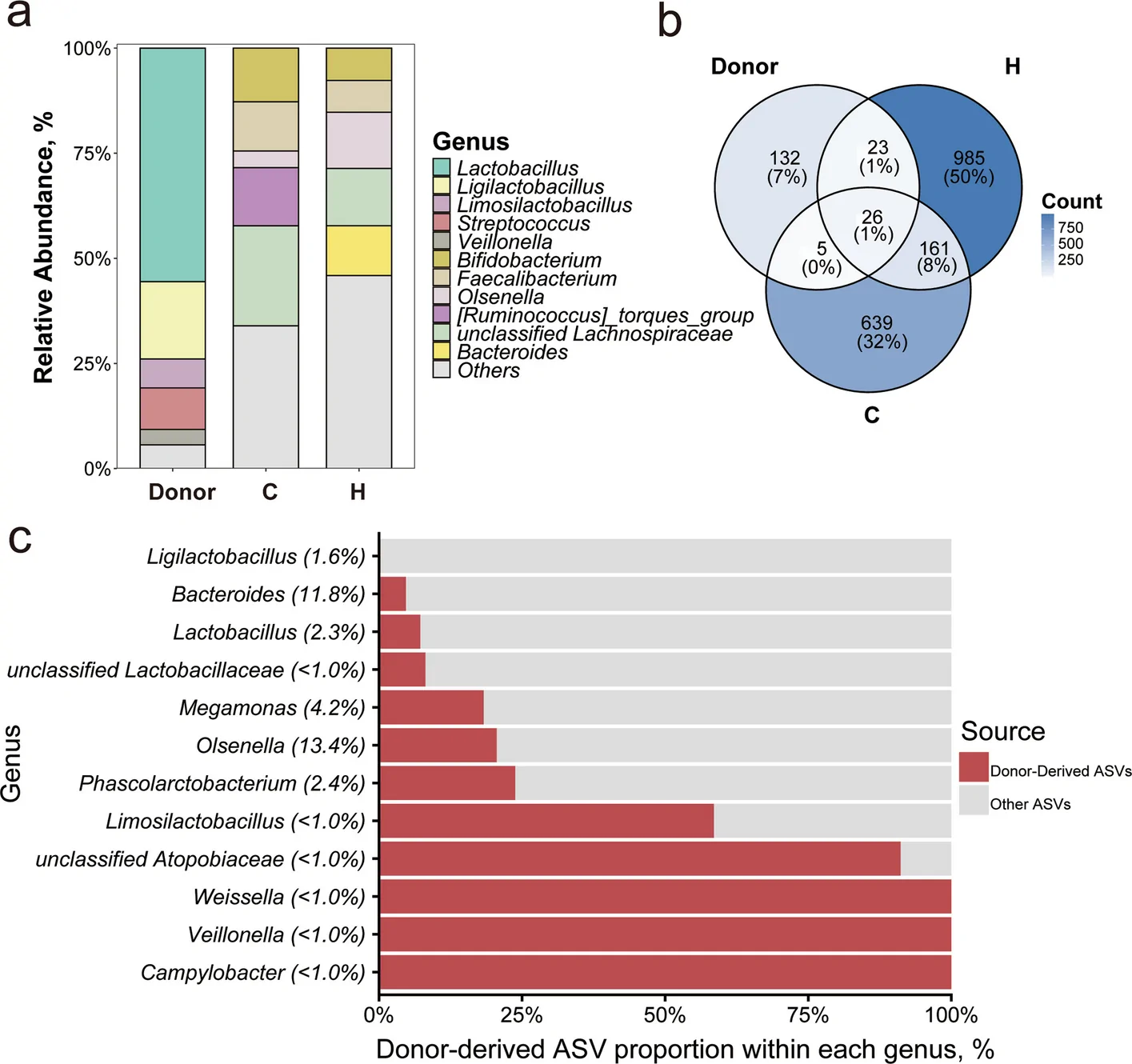
Donor fecal microbial transmission and cecal microbiota composition in broilers. **a** Relative abundance of the top 5 genera within each respective group in the donor feces and D 10 cecal contents of the C and H groups. **b** Venn diagram showing shared and unique Amplicon Sequence Variants (ASVs) among the Donor, C, and H groups. **c** Proportion of donor-derived ASVs within each genus in the H group. The *y*-axis lists the genera detected in the H-group cecal microbiota, with percentages in parentheses indicating the relative abundance of each genus. The *x*-axis indicates the proportion of donor-derived ASVs within each respective genus (%). Donor-derived ASVs were defined as ASVs shared exclusively between the donor and H groups but absent from the C group. These ASVs were assigned to their corresponding genera. For each genus, the donor-derived proportion was calculated as the summed abundance of donor-derived ASVs divided by the total abundance of that genus in the H group. C: Control; H: Microbial contact. *n* = 4 for donor feces; *n* = 8 for cecal content of broilers

As shown in Fig. 2a and Additional file 1: Table S4, in donor’s fecal microbiota, the top five dominant genera were *Lactobacillus*, *Ligilactobacillus*, *Streptococcus*, *Limosilactobacillus*, and *Veillonella*. On D 10, for group C, the most abundant cecal genera included unclassified *Lachnospiraceae*, the *[Ruminococcus]_torques_group*, *Bifidobacterium*, *Faecalibacterium*, and *Olsenella*. In group H, the top five genera were unclassified *Lachnospiraceae*, *Olsenella*, *Bacteroides*, *Bifidobacterium*, and *Faecalibacterium*. A total of 1,971 ASVs were identified across the donor, recipient, and control groups. A Venn diagram (Fig. 2b) revealed distinct microbial profiles, with the recipient group harboring the highest number of unique ASVs (*n* = 985). Notably, 23 ASVs were exclusively shared between the donor and recipient groups, whereas only 5 ASVs were shared exclusively between the donor and control groups. 26 ASVs were shared among all three groups. Taxonomic annotation of the 23 ASVs shared between the donor and recipient identified 12 distinct genera (Fig. 2c). The ASVs assigned to *Campylobacter*, *Veillonella*, and *Weissella* in the recipient group were exclusively shared with the donor, being absent in the control group. Similarly, within their respective genera, these donor-shared ASVs accounted for the majority of the total relative abundance for unclassified *Atopobiaceae* (91%) and *Limosilactobacillus* (59%). In contrast, within the remaining shared genera, the relative abundance of donor-shared ASVs represented less than 25% of the total genus abundance.

To further compare the donor hen fecal microbiota with the cecal microbiota of D 10 broilers, alpha- and beta-diversity analyses were performed (Additional file 2: Fig. S1). The H group exhibited significantly higher Chao1 and Shannon indices than the donor group (*P* < 0.05), whereas neither index differed significantly between the donor and C groups (*P* > 0.05). Bray-Curtis PCoA further demonstrated significant differences in microbial community composition among the donor, C, and H groups (overall PERMANOVA, *P* < 0.01; all pairwise PERMANOVA comparisons, *P* < 0.01).

#### Alpha diversity of broiler cecal microbiota

**Fig. 3:**
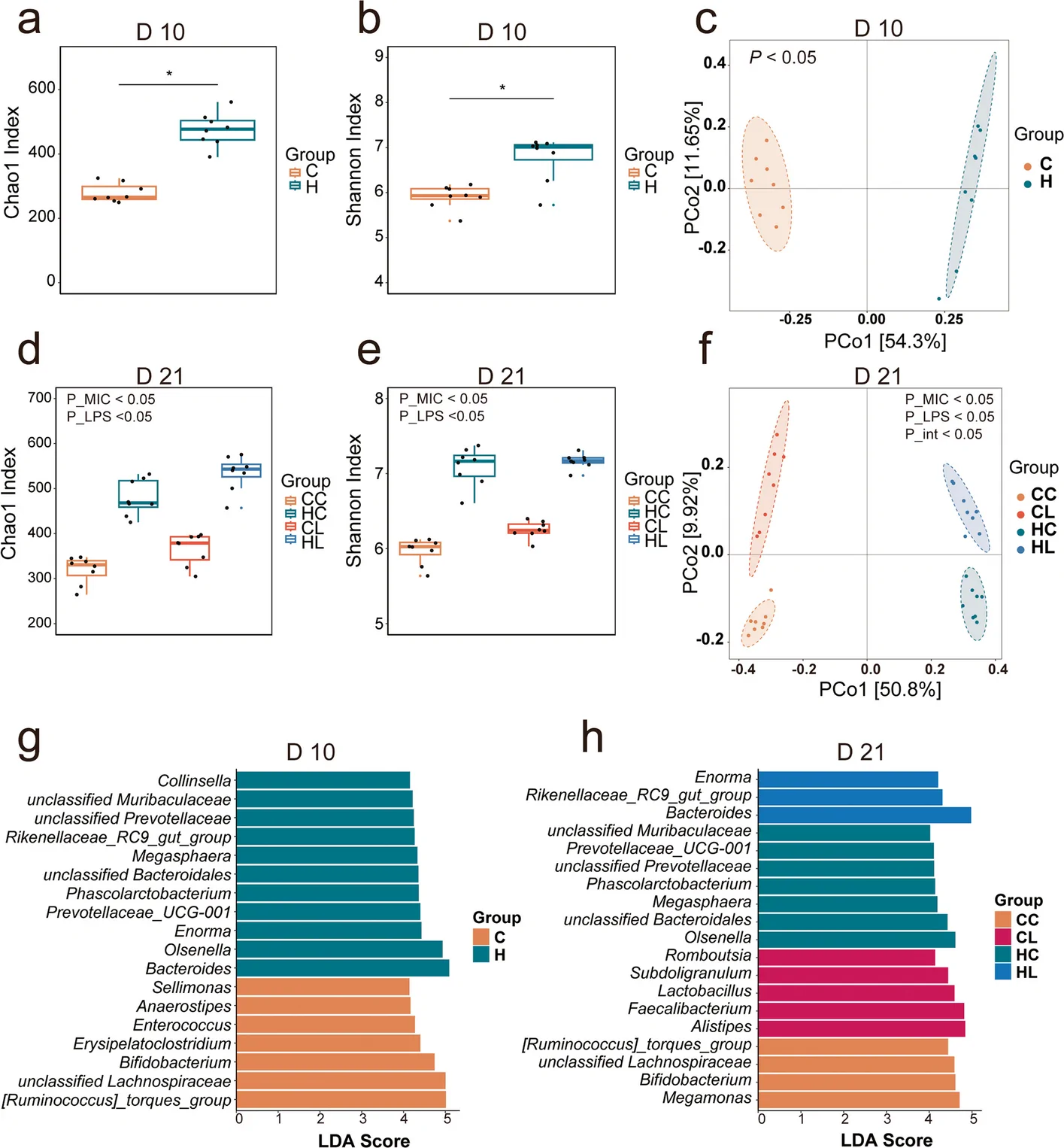
Effects of microbial contact on microbial diversity and differential abundance of genera. **a**, **b**, **d**, **e** Chao1 and Shannon indices of the cecal microbiota in broilers. **c**, **f** Beta diversity of the cecal microbiota based on Bray-Curtis distance. **g**, **h** Linear discriminant analysis Effect Size (LEfSe) identifying differentially abundant genera in the cecal microbiota. C: control; H: microbial contact; CC: control + PBS; HC: microbial contact + PBS; CL: control + LPS; HL: microbial contact + LPS. *n* = 8 biological replicates. For D 10, data were analyzed using Student’s *t*-test (^*^P < 0.05). For D 21, a two-way ANOVA was employed to evaluate the main effects of microbial contact (*P*_MIC) and LPS (*P*_LPS) challenge, as well as their interaction (*P*_int) on alpha diversity, while PERMANOVA was used to evaluate these effects on beta diversity. Significance was defined as *P* < 0.05. LEfSe was performed with a threshold of |LDA| > 4 and adjusted *P* < 0.05

On D 10, group H exhibited significantly higher Chao1 and Shannon indices compared to group C (Fig. 3a and b; *P* < 0.05). On D 21, both the microbial contact (*P* < 0.05) and LPS (*P* < 0.05) significantly increased the Chao1 and Shannon indices, but no significant interaction was observed between them (*P* = 0.724 and *P* = 0.082, respectively; Fig. 3d and e).

#### Beta diversity of broiler cecal microbiota

On D 10, β-diversity analysis based on Bray-Curtis distances revealed significant differences between the C and H groups (Fig. 3c; *P* < 0.05).

On D 21, both the microbial contact and the LPS challenge significantly altered the microbial community structure (Fig. 3f). The microbial contact explained 50% (*R*^2^ = 0.50) of the total variation (*P_*MIC < 0.05). The LPS challenge also had a significant but smaller effect (*R*^2^ = 0.09, *P_*LPS < 0.05). Notably, a significant interaction between the microbial contact and the LPS challenge was observed (*R*^2^ = 0.06, *P_*int < 0.05), indicating that the impact of LPS on microbial composition depended on the microbiota background.

#### Differentially abundant genera

On D 10 (Fig. 3g), LEfSe analysis identified 7 differentially enriched genera in the C group, including the *[Ruminococcus]_torques_group*, unclassified *Lachnospiraceae*, *Bifidobacterium*, *Erysipelatoclostridium*, *Enterococcus*, *Anaerostipes*, and *Sellimonas*. Conversely, the H group was characterized by the enrichment of 11 genera, including *Bacteroides*, *Olsenella*, *Enorma*, *Prevotellaceae UCG-001*, *Phascolarctobacterium*, unclassified *Bacteroidales*, *Megasphaera*, the *Rikenellaceae_RC9_gut_group*, unclassified *Prevotellaceae*, unclassified *Muribaculaceae*, and *Collinsella*.

On D 21 (Fig. 3h), distinct microbial signatures were identified for each treatment group. The CC group was characterized by *Megamonas*, *Bifidobacterium*, unclassified *Lachnospiraceae*, and the *[Ruminococcus]_torques_group*. The CL group was associated with *Alistipes*, *Faecalibacterium*, *Lactobacillus*, *Subdoligranulum*, and *Romboutsia*. The HC group was enriched with *Olsenella*, unclassified *Bacteroidales*, *Megasphaera*, *Phascolarctobacterium*, unclassified *Prevotellaceae*, *Prevotellaceae UCG-001*, and unclassified *Muribaculaceae*. The HL group was associated with *Bacteroides*, the *Rikenellaceae_RC9_gut_group*, and *Enorma*.

### Metabolites

#### Volatile fatty acids in cecum

**Fig. 4:**
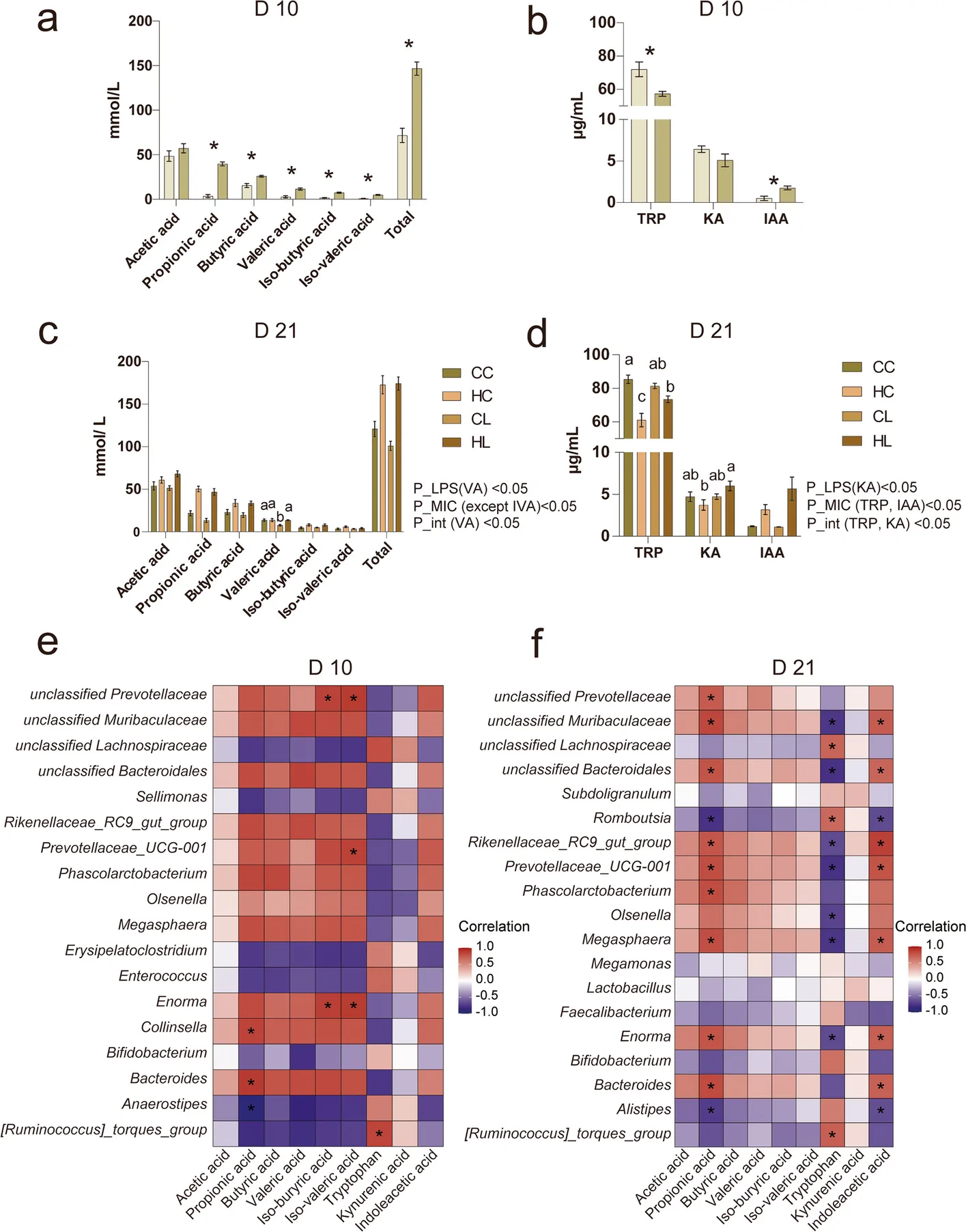
Effects of microbial contact on cecal metabolite profiles and microbiota-metabolite correlations. **a**, **b** Cecal volatile fatty acid (VFA) concentrations (mmol/L) and serum tryptophan (TRP), kynurenic acid (KA), and indoleacetic acid (IAA) concentrations (μg/mL) on D 10. **c**, **d** Corresponding cecal VFA and serum tryptophan metabolite concentrations on D 21. **e**, **f** Heatmaps of Spearman’s rank correlations between differentially abundant genera and metabolite concentrations on D 10 and D 21, respectively. C: control; H: microbial contact; CC: control + PBS; HC: microbial contact + PBS; CL: control + LPS; HL: microbial contact + LPS (*n* = 8 biological replicates). VA: valeric acid; IVA: iso-valeric acid. Data on D 10 were analyzed using Student’s *t*-test (*P* < 0.05). Data on D 21 were evaluated using a two-way ANOVA to assess the main effects of microbial contact (*P*_MIC) and LPS challenge (*P*_LPS), as well as their interaction (*P*_int). For significant interactions (*P* < 0.05), Tukey’s post-hoc test was performed, where different letters indicate significant pairwise differences of the interaction effects. In the Spearman heatmaps, asterisks denote significant correlations (|*R*| > 0.5, adjusted *P* < 0.01)

On D 10 (Fig. 4a), group H exhibited significantly higher concentrations of propionic acid (PA), butyric acid (BA), valeric acid (VA), iso-butyric acid (IBA), iso-valeric acid (IVA), and total VFAs compared to group C (*P* < 0.05).

On D 21 (Fig. 4c), the LPS challenge significantly decreased the concentration of VA (*P_*LPS < 0.05). The microbial contact significantly increased the concentrations of all measured VFAs, except for IVA (*P_*MIC < 0.05). A significant interaction between the microbial contact and LPS challenge was observed for VA (*P_*int < 0.05). Specifically, the concentration of VA in the CL group was significantly lower than that in the CC, HC, and HL groups (*P* < 0.05), while no significant differences were observed among the latter three groups.

#### Tryptophan metabolites in serum

On D 10 (Fig. 4b), group H exhibited significantly lower serum TRP levels and higher serum indoleacetic acid (IAA) levels compared to group C (*P* < 0.05).

On D 21 (Fig. 4d), the LPS challenge significantly increased serum kynurenic acid (KA) levels (*P_*LPS < 0.05). The microbial contact significantly reduced serum TRP levels and increased IAA levels (*P_*MIC < 0.05). Significant interaction effects between the microbial contact and LPS challenge were observed for TRP and KA (*P_*int < 0.05). Specifically, serum TRP levels in the HL group were significantly lower than those in the CC group but significantly higher than those in the HC group (*P* < 0.05). Serum KA levels in the HL group were significantly higher than those in the HC group (*P* < 0.05).

### Spearman correlations of key genera with host metabolites

On D 10 (Fig. 4e), *Anaerostipes* showed a strong negative correlation with PA levels. In contrast, *Bacteroides* and *Collinsella* were positively correlated with this PA. IBA was positively associated with *Enorma* and unclassified *Prevotellaceae*. Similarly, IVA showed strong positive correlations with *Enorma*, unclassified *Prevotellaceae*, and *Prevotellaceae UCG-001*. Additionally, TRP levels displayed a significant positive correlation with the *[Ruminococcus]_torques_group*.

On D 21 (Fig. 4f), PA was positively correlated with *Bacteroides*, *Enorma*, *Megasphaera*, *Phascolarctobacterium*, *Prevotellaceae UCG-001*, *Rikenellaceae_RC9_gut_group*, as well as unclassified *Bacteroidales*, *Muribaculaceae*, and *Prevotellaceae*, while showing negative correlations with *Alistipes* and *Romboutsia*. Similarly, IAA showed positive associations with *Bacteroides*, *Enorma*, *Megasphaera*, *Prevotellaceae UCG-001*, *Rikenellaceae_RC9_gut_group*, unclassified *Bacteroidales*, and unclassified *Muribaculaceae*, but was negatively correlated with *Alistipes* and *Romboutsia*. Conversely, TRP levels were positively linked to the *[Ruminococcus]_torques_group*, *Romboutsia*, and unclassified *Lachnospiraceae*, whereas negative correlations were observed with *Enorma*, *Megasphaera*, *Olsenella*, *Prevotellaceae UCG-001*, *Rikenellaceae_RC9_gut_group*, unclassified *Bacteroidales*, and unclassified *Muribaculaceae*.

### Gene expression

**Fig. 5:**
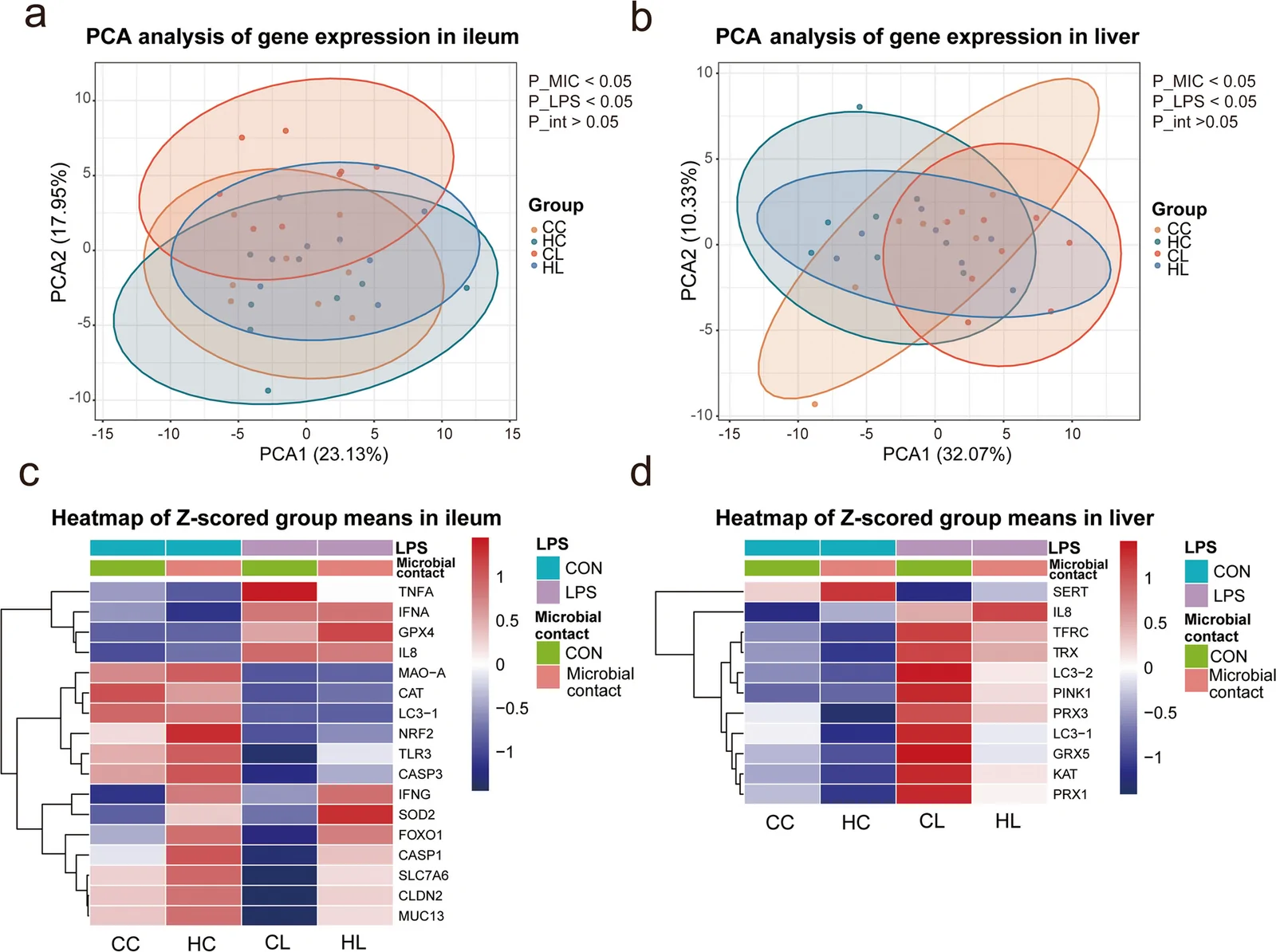
PCA and heatmaps of targeted gene expression profiles in the ileum and liver on D 21. **a** and **b** PCA plots of the ileal (**a**) and hepatic (**b**) overall gene expression profiles based on Euclidean distance. The displayed *P*-values indicate the main effects of microbial contact (*P*_MIC) and LPS challenge (*P*_LPS), as well as their interaction (*P*_int) on overall expression patterns, as evaluated using PERMANOVA. **c** and **d** Heatmaps illustrating the gene expression patterns in the ileum (**c**) and liver (**d**). To construct the heatmaps, genes were strictly filtered to include only those demonstrating a significant main effect based on a two-way ANOVA with Benjamini-Hochberg FDR correction (adjusted *P* < 0.05). Data are visualized as Z-scored group means. CC: control + PBS; HC: microbial contact + PBS; CL: control + LPS; HL: microbial contact + LPS (*n* = 8 biological replicates). *CASP1* Caspase 1, *CASP3* Caspase 3, *CAT* Catalase, *CLDN2* Claudin 2, *FOXO1* Forkhead box O1, *GPX4* Glutathione peroxidase 4, *GRX5* Glutaredoxin 5, *IFNA* Interferon alpha, *IFNG* Interferon gamma, *IL8* Interleukin 8, *KAT* Kynurenine aminotransferase, *LC3-1* Microtubule-associated protein 1 light chain 3 alpha, *LC3-2* Microtubule-associated protein 1 light chain 3 beta, *MAO-A* Monoamine oxidase A, *MUC13* Mucin 13, *NRF2* Nuclear factor erythroid 2-related factor 2, *PINK1* PTEN induced kinase 1, *PRX1* Peroxiredoxin 1, *PRX3* Peroxiredoxin 3, *SERT* Serotonin transporter, *SLC7A6* Solute carrier family 7 member 6, *SOD2* Superoxide dismutase 2, *TFRC* Transferrin receptor, *TLR3* Toll-like receptor 3, *TNFA* Tumor necrosis factor alpha, *TRX *Thioredoxin

A total of 86 genes in the ileum on D 21 were evaluated to assess their role in host physiological responses (Additional file 1: Table S5). Two-way PERMANOVA based on Euclidean distances revealed significant main effects for both microbial contact (*R*^2^ = 0.07, *P_*MIC = 0.009) and LPS challenge (*R*^2^ = 0.10, *P_*LPS = 0.001) on the ileal gene expression profiles (Fig. 5a). However, no significant interaction effect was observed between microbial contact and LPS (*P_*int = 0.314), suggesting that the modulation of gene expression by microbial contact was independent of the LPS exposure status. As visualized in the heatmap (Fig. 5c) and detailed in Additional file 1: Table S5, on D 21, the microbial contact significantly (*P* < 0.05) upregulated the relative expression of ileal mucin 13 (*MUC13*), claudin 2 (*CLDN2*), forkhead box protein O1 (*FOXO1*), interferon gamma (*IFNG*), caspase 1 (*CASP1*), and superoxide dismutase 2 (*SOD2*). Conversely, the LPS challenge significantly (*P* < 0.05) downregulated the expression of catalase (*CAT*), *MUC13*, caspase 3 (*CASP3*), monoamine oxidase A (*MAO-A*), *CLDN2*, nuclear factor erythroid 2-related factor 2 (*NRF2*), solute carrier family 7 member 6 (*SLC7A6*), microtubule-associated protein 1 light chain 3 alpha (*LC3-1*), and Toll-like receptor 3 (*TLR3*), while significantly (*P* < 0.05) upregulating glutathione peroxidase 4 (*GPX4*), interferon alpha (*IFNA*), interleukin 8 (*IL8*), and tumor necrosis factor alpha (*TNFA*).

A total of 83 genes in the liver on D 21 were evaluated to assess their role in host physiological responses (Additional file 1: Table S6). Both microbial contact (*R*^2^ = 0.08, *P*_MIC = 0.018) and LPS challenge (*R*^2^ = 0.10, *P_*LPS = 0.003) significantly influenced the overall gene expression profiles (Fig. 5b). Additionally, the interaction effect between microbial contact and LPS was also not statistically significant (*P_*int = 0.357). As visualized in the heatmap (Fig. 5d) and detailed in Additional file 1: Table S6, the microbial contact significantly (*P* < 0.05) downregulated glutaredoxin 5 (*GRX5*) and *LC3-1*. The LPS challenge significantly (*P* < 0.05) upregulated the expression of *GRX5*, *IL8*, kynurenine aminotransferase (*KAT*), *LC3-1*, *LC3-2*, PTEN-induced kinase 1 (*PINK1*), peroxiredoxin 1 (*PRX1*), peroxiredoxin 3 (*PRX3*), transferrin receptor (*TFRC*), and thioredoxin (*TRX*), while significantly (*P* < 0.05) downregulating serotonin transporter (*SERT*).

## Discussion

The maternal microbiota plays a pivotal role in shaping the early-life microbial development of the offspring [4, 7, 8], and the appropriate colonization of the gut microbiota is essential for enhancing immune function [7]. However, current commercial broiler production practices disrupt the vertical transmission of microbiota from hens to chicks [6]. Accordingly, this study investigated the effects of adult hen microbial contact on growth performance, cecal microbiota composition, and resilience against immunological stress in broilers.

Adult hen microbial contact did not significantly affect body weight or ileal histomorphology, consistent with previous microbial intervention studies [12, 30]. This suggests that the benefits of early-life microbial exposure may not manifest as short-term growth enhancement, but rather through modulation of gut microbiota and host physiology. The lack of growth response is likely donor-dependent, as the efficacy of such interventions is known to be shaped by the composition and functional attributes of the donor microbiota [6]. Similarly, while LPS is widely used to induce intestinal inflammation and immune stress in broilers, the present challenge did not cause significant weight suppression, corroborating earlier reports [20, 31]. Instead, the LPS model primarily provoked physiological and molecular alterations, making it appropriate for assessing intervention effects on host resilience [20]. Notably, no significant interactions between microbial contact and LPS challenge were observed for either body weight or ileal histomorphology. Together, these results imply that the protective effects of microbial contact are unlikely to be mediated via improved growth performance or intestinal morphology, but may instead operate through shifts in microbial composition, metabolic activity, and host physiological responses.

We observed that while microbial contact significantly influenced the composition of the chick gut microbiota, this impact was not primarily driven by the direct colonization of donor fecal genera within the recipients. Although *Lactobacillus* was the most predominant genus in the donor hen feces, donor-derived *Lactobacillus* contributed only minimally to the recipient microbiota. This suggests that high abundance in the donor does not guarantee efficient colonization in the chick gut, likely due to the specific ecological requirements necessary for bacterial establishment [6]. In contrast, *Bacteroides* became highly enriched in the recipient microbiota despite only limited overlap with the donor. This observation suggests that the enrichment of *Bacteroides* likely originated from environmental sources rather than via direct transmission from the donor fecal microbiota. While previous studies have suggested that donor feather, oral, and skin microbiota can contribute to colonization, their influence is generally minor compared to that of the donor fecal microbiota [4, 8]. A more plausible explanation is that the pioneer species from the donor feces rapidly occupied the chick’s gut niche, modifying the gut environment to favor the recruitment and colonization of other commensals from the environment [5, 6]. This ecological succession model is further supported by the successful establishment of *Olsenella*, which occupied an important ecological niche despite its relatively low abundance in the donor. Given that *Olsenella* is recognized as a beneficial genus positively correlated with growth performance [32], its successful colonization underscores the potential value of the microbial contact. Interestingly, several genera, including *Campylobacter*, *Veillonella*, and *Weissella*, were detected exclusively in recipients exposed to microbial contact, indicating that direct microbial transmission did occur. However, their consistently low abundance suggests that direct colonization contributed only modestly to the overall microbial community structure. Similar studies have reported the vertical transmission of *Campylobacter* and *Veillonella* from hens to chicks primarily through early-life fecal contact, though the extent of their colonization abundance in recipients has not been previously quantified [4, 7]. Furthermore, it is noteworthy that both the donor hens and the recipient chicks were provided with the same standard broiler diet. We speculate that the absence of specific dietary substrates in this feed may have limited the establishment of certain donor-derived taxa, although this needs further experimental verification. This speculation is consistent with previous findings that supplementing early-life diets with specific prebiotics, such as inulin, can enhance colonization efficiency following early fecal contact [33]. Taken together, we reason that integrating microbial-nutritional strategies may therefore represent a practical approach to optimize early-life gut microbiota development in commercial broiler production.

In the present study, microbial contact significantly increased alpha diversity, and this diversity advantage persisted until D 21. Similarly, a significant elevation in alpha diversity among recipients has been widely reported in various microbial intervention trials [4, 11]. Although increased alpha diversity alone should not be interpreted as an indicator of a healthier microbial community [34], the increase observed in the present study was accompanied by the enrichment of beneficial microbiota and enhanced microbial metabolite production, which likely reflects enhanced microbial community maturation. In contrast, the LPS challenge also independently increased alpha diversity, consistent with prior studies [19, 35]. Unlike microbial contact, this increase has been attributed to disruption of the intestinal microbial ecosystem under inflammatory conditions rather than beneficial microbial maturation [35, 36]. Furthermore, despite the relatively low quantitative abundance of genera directly shared with donor feces, the persistent separation in beta diversity demonstrated that microbial contact exerted long-lasting effects on microbial community structure. While the LPS challenge also induced significant structural perturbations in the microbial community, consistent with previous models of immunological stress [37, 38], its impact was quantitatively less pronounced than that of the microbial contact, as evidenced by the effect sizes in our statistical models. These findings suggest that early microbial intervention may help establish a gut microbial community that responds differently to subsequent immune challenges [6].

The microbial biomarkers identified in the H group were predominantly associated with two beneficial functional categories, namely polysaccharide degradation and VFA production. *Bacteroides*, *Prevotellaceae*, and *Rikenellaceae* are recognized as primary degraders of dietary fibers and non-starch polysaccharides [6, 13, 39], whereas *Phascolarctobacterium* and *Megasphaera* further convert fermentation intermediates into VFAs through cross-feeding interactions [40, 41]. These cross-feeding interactions likely explain the increased VFA concentrations observed following microbial contact, underscoring the importance of early microbial exposure in promoting gut health through microbial metabolite production [14]. While previous studies have reported that VFAs stimulate epithelial proliferation and improve intestinal morphology [14], no significant alterations in ileal histomorphology were observed in the present study. We speculate that these metabolic changes may have contributed more to microbial homeostasis and immune function than to structural intestinal development. This interpretation is further supported by the LPS challenge model, in which LPS significantly reduced valeric acid concentrations, whereas microbial contact largely preserved valeric acid concentrations. Given that valeric acid has been widely used as a feed additive to alleviate intestinal inflammation [42], its preservation may contribute to maintaining microbial metabolic function during immune stress. Furthermore, the microbial contact consistently reduced serum TRP while increasing IAA levels, suggesting enhanced microbial conversion of TRP into indole derivatives [15]. This finding is consistent with positive correlations between IAA and several enriched polysaccharide-degrading genera, including *Bacteroides*, *Rikenellaceae*, *Prevotellaceae*, and *Muribaculaceae*. These genera are known to express tryptophanase, which converts TRP into indole derivatives, including IAA [15, 17]. The generated IAA acts as a ligand for the aryl hydrocarbon receptor and plays a pivotal role in strengthening the intestinal barrier and regulating mucosal immunity [15, 17]. Additionally, microbial contact increased serum kynurenic acid following LPS challenge, suggesting enhanced activation of the kynurenine pathway during immune stress. Because efficient TRP metabolism depends on an established gut microbiota [43], these findings indicate that early microbial contact improved microbial metabolic adaptation to subsequent immune challenge. Collectively, these findings suggest that early microbial intervention may represent a practical strategy to improve microbial metabolic function and maintain microbial eubiosis in commercial broiler production. Future studies should quantify these metabolites directly in the cecum and combine shotgun metagenomics with metabolomics to validate the functional activities of these microbial pathways.

To elucidate the role of microbial metabolites in host-microbiota cross-talk, we investigated their impact on ileal and hepatic gene regulation. The microbial contact significantly altered the overall gene expression profile of the ileum, primarily by improving the ileal barrier function and antioxidant defenses. Regarding barrier function, the microbial contact enhanced the expression of key genes involved in mucosal protection and epithelial integrity, including *MUC13* and *CLDN2* [44]. This upregulation is likely driven by the elevated concentrations of VFAs and IAA observed in the microbial contact group, which are known to transcriptionally upregulate these mucosal defense proteins [14, 15, 38]. Concurrently, the microbial contact enhanced the host antioxidant defence, as evidenced by the elevation of the key antioxidant regulators *SOD2* and *FOXO1* [18]. In contrast, the LPS challenge caused a suppression of the antioxidant system, characterized by the downregulation of *CAT* and *NRF2*, and triggered an inflammatory response (*IL8*, *TNFA*, *IFNα*) [45]. Collectively, these transcriptional changes suggest that LPS impaired intestinal barrier integrity and nutrient absorptive capacity, as reflected by the downregulation of *MUC13*, *CLDN2*, and *SLC7A6* [44, 46]. These findings align with previous research demonstrating that LPS significantly increases reactive oxygen species while depleting antioxidant reserves in chickens, leading to intestinal damage [18, 20, 35]. In the liver, both the microbial contact and the LPS challenge significantly altered the gene expression profiles. As expected, the LPS challenge upregulated mitochondrial antioxidant genes (*GRX5*, *PRX1*, *PRX3*, *TRX*) and autophagy markers (*LC3-1*, *LC3-2*). This concerted upregulation reflects a compensatory hepatic response triggered to combat LPS-induced oxidative stress and cellular damage [47–50], a finding also reported in other chicken LPS challenge trials [51, 52]. Conversely, the microbial contact exhibited a directly opposite pattern, characterized by the significant downregulation of *GRX5* and *LC3-1*. Given the absence of a significant interaction between the two factors, this downregulation suggests that early-life microbial contact may reduce the activation of hepatic antioxidant and autophagic pathways under physiological conditions, potentially contributing to the maintenance of hepatic homeostasis. Collectively, these hepatic responses should be interpreted within a gut-liver axis framework [53]. We propose that microbial contact elevates gut-derived metabolites, including VFAs and serum tryptophan metabolites. These circulating signals sustain intestinal barrier integrity and reduce the transfer of pro-inflammatory triggers to the liver upon LPS exposure [20, 50, 53]. Consequently, the liver requires less activation of antioxidant and autophagy pathways to counteract stress, consistent with the divergent transcriptional changes observed between the LPS challenge and microbial contact treatments.

A couple of limitations of this study should be acknowledged. First, we did not include an adult broiler donor group, which prevents us from determining whether the observed effects were due to exposure to a mature adult microbiota in general or were specific to the ISA Brown laying hen donors used here. Future studies should compare different breeds of chickens as donors to determine whether donor breed influences microbial establishment and host responses. Second, only one donor hen was assigned to each microbial-contact pen, meaning that donor‑specific effects were confounded with pen‑level variation. This may have introduced variability in recipient microbial development across pens. Although we accounted for pen‑level variance by including pen as a random effect in the linear mixed model, future studies could implement a rotational housing strategy, in which donor hens are periodically swapped among pens, to minimize the impact of inter‑individual donor variation on recipient microbiota. Third, our study reared the animals only up to 21 days. Future studies should follow up the animals and their performance up to slaughter age.

## Conclusion

In conclusion, our study demonstrates that early exposure to the adult hen microbiota significantly changed the colonization of the broiler gut microbiota. Importantly, this modulation was not driven by simple direct transmission, but rather by the selective colonization of ecologically dominant and functionally significant genera, including *Bacteroides*, *Prevotellaceae*, *Rikenellaceae*, *Muribaculaceae*, and *Megasphaera*. This structural shift reshaped the intestinal metabolite landscape, characterized by the enhanced production of VFA and the TRP metabolite IAA, both being critical for enhancing intestinal barrier function. Notably, microbial contact attenuated the LPS-induced reduction in VA, highlighting its role in preserving microbial metabolite output under immunological stress. Host gene expression analysis further corroborated these functional improvements, evidencing strengthened mucosal barriers and enhanced antioxidant capacity in the microbial contact group. Collectively, this study establishes that restoring natural microbial contact is a potent strategy to modulate microbiota composition, thereby enhancing intestinal barrier integrity and modulating metabolite profiles to improve broiler tolerance to subsequent immune stress. Nevertheless, these findings are derived from a 21‑day short‑term trial, and the long-term persistence of these effects and their applicability across different donor breeds remain to be determined. Future studies should further explore the practical application of adult hen microbial contact in commercial broiler production, particularly in combination with targeted prebiotic supplementation to optimize early-life gut microbiota establishment and function. Additionally, translation to large‑scale industrial settings will need to address the practical delivery of complex microbiota and consider the regulatory context governing their application.

## Supplementary Information

Additional file 1: Table S1. Composition of the feed offered to broilers during starter (D 0–D 10) and grower phases (D 10–D 21). Table S2. Selected genes panel of ileum to investigate gut health of broiler chickens. Table S3. Selected genes panel of liver to investigate host health of broiler chickens. Table S4. Fecal microbiota composition of the four individual ISA Brown donor hens at the start of the experiment. Table S5. Effects of microbial contact and LPS challenge on ileal gene expression profiles. Table S6. Effects of microbial contact and LPS challenge on hepatic gene expression profiles.

Additional file 2: Fig. S1. Alpha and beta diversity of donor hen fecal microbiota and D 10 broiler cecal microbiota.

## Data Availability

The 16S rRNA gene sequencing data can be accessed under Bioproject accession PRJNA1452812 at the NCBI website.

## Abbreviations

ASV: Amplicon Sequence Variant
BA: butyric acid
CD: crypt depth
FDR: False Discovery Rate
IAA: indoleacetic acid
IBA: iso-butyric acid
IFCs: integrated fluidic circuits
IVA: iso-valeric acid
KA: kynurenic acid
LEfSe: Linear Discriminant Analysis Effect Size
LPS: lipopolysaccharide
PA: propionic acid
PBS: phosphate-buffered saline
PCA: Principal Component Analysis
PCoA: Principal Coordinate Analysis
PDA: photodiode array
TRP: tryptophan
V/C: villus height to crypt depth ratio
VA: valeric acid
VFA: volatile fatty acid
VH: villus height
