# Supplementary material for: Early-life microbial contact with adult hens modulates cecal microbiota and mitigates host responses to lipopolysaccharide-induced stress in broilers
Source: J Anim Sci Biotechnol. 2026 Oct 10;17:192. doi: 10.1186/s40104-026-01511-x (PMC13653393; doi:10.1186/s40104-026-01511-x)
Supplement: Supplementary file 1 [file 40104_2026_1511_MOESM2_ESM.docx]

Fig. S1. Alpha and beta diversity of donor hen fecal microbiota and D 10 broiler cecal microbiota. (a) Chao1 index and (b) Shannon index of the control (C), microbial contact (H), and donor groups. The H group showed significantly higher Chao1 and Shannon indices than the C group (*P* = 0.007 and *P* = 0.035, respectively) and the donor group (*P* < 0.001), whereas no significant differences were observed between the C and donor groups (*P* > 0.05). (c) Principal coordinate analysis based on Bray-Curtis distances showed clear separation among the three groups, with significant differences in microbial community composition for all pairwise comparisons (overall PERMANOVA, *P* < 0.01; all pairwise PERMANOVA comparisons, *P* < 0.01). Each point represents one biological replicate, and ellipses indicate 95% confidence intervals. *n* = 4 for Donor and *n* = 8 for C and H. ns, not significant; **P* < 0.05.
